# Vaccine Efficacy of Bm86 Ortholog of *H. a. anatolicum*, rHaa86 Expressed in Prokaryotic Expression System

**DOI:** 10.1155/2009/165812

**Published:** 2010-02-08

**Authors:** P. Azhahianambi, D. D. Ray, Pallab Chaudhuri, Rohita Gupta, Srikanta Ghosh

**Affiliations:** ^1^Centre for Biosystem Research, University of Maryland Biotechnology Institute, Rockville, MD 20742-4450, USA; ^2^Entomology Laboratory, Parasitology Division, Indian Veterinary Research Institute, Izatnagar, 243122 Uttar Pradesh, India; ^3^Division of Bacteriology and Mycology, Indian Veterinary Research Institute, Izatnagar, 243122 Uttar Pradesh, India

## Abstract

The use of tick vaccine in controlling ticks and tick borne diseases has been proved effective in integrated tick management format. For the control of *H. a. anatolicum*, Bm86 ortholog of *H. a. anatolicum* was cloned and expressed as fusion protein in *E. coli* as *E. coli*-pETHaa86. The molecular weight of the rHaa86 was 97 kDa with a 19 kDa fusion tag of thioredoxin protein. The expressed protein was characterized immunologically and vaccine efficacy was evaluated. After 120 hours of challenge, only 26% tick could successfully fed on immunized animals. Besides significant reduction in feeding percentages, a significant reduction of 49.6 mg; *P* < .01 in the weight of fed females in comparison to the females fed on control animals was recorded. Following oviposition, a significant reduction of 68.1 mg; *P* < .05 in the egg masses of ticks fed on immunized animals in comparison to the ticks fed on control animals was noted. The reduction of number of females, mean weight of eggs, adult females and efficacy of immunogen were 73.8%, 31.3%, 15.8%, and 82.3%, respectively. The results indicated the possibility of development of rHaa86 based vaccine as a component of integrated control of tick species.

## 1. Introduction

The three-host tick, *Hyalomma a. anatolicum*, is one of the most widely distributed tick species of India infesting cattle, buffaloes, sheep and goat and transmitting *Theileria annulata, T. buffeli, T. lestocardi* (*T. hirci*) [1, 2]. The predicated distribution of bovine tropical theileriosis in India and cattle and buffalo population at risk is well documented [3]. Recently, the control cost ticks and the parasites they transmit have been roughly estimated in the tune of 441.5 million US$/annum. Besides the vectorial potential of the tick species, the significant direct effect of ticks on livestock production necessitated to develop tick control methods in an environmentally safe manner.

In India tick control is focused on repeated direct application of acaricides on host animals. In most of the cases the application of acaricides is repeated after 21 to 30 days. The approach has limitations including development of acaricide resistance, environmental contamination, pesticide residues in food products and the expense of developing new pesticides [4]. Other tick control method which shows promise is the use of anti-tick vaccines [5–7]. Although there were some problems associated with commercialization of the tick vaccines developed against cattle tick, *Boophilus microplus *[7], the success of the present tick vaccine (TickGARD Plus and Gavac) in the past ten years has clearly demonstrated their potential as an effective method of tick control [8]. 

In the case of *H. a. anatolicum*, some potential antigens have been identified and tested against experimental challenge infestations and the subject has been reviewed by Ghosh et al. [9] but the work has not been reached at the level of development of vaccine against *H. a. anatolicum*. On the other hand, although the homologue of Bm86 has been reported in *H. a. anatolicum* [10], the heterologous BM86 based vaccine has limited efficacy against experimental challenge of *H. a. anatolicum*. Recently, Bm86 ortholog of *H. a. anatolicum* has been cloned and expressed in *Pichia pastoris* and was found protective against homologous challenge infestation [11]. However, expression of the recombinant protein was low. With a target to increase the expression level and to reduce the steps of purification, the present experiment was undertaken to clone and express the Bm86 ortholog of *H. a. anatolicum *in prokaryotic expression system and to analyze the protective efficacy of the expressed protein against homologous challenge infestations. 

## 2. Material and Methods

### 2.1. Rabbits

New Zealand white rabbits, weighing approximately 1 to 1.5 kg were obtained from Laboratory Animal Resource section of IVRI, Izatnagar. They were maintained in disinfected cages of small animal house of the Division of Parasitology and were fed *ad libitum.* Rabbits were used for rearing of *Theileria annulata* free* H. a. anatolicum* and also to raise specific antibodies.

### 2.2. Cross-Bred Calves

 Thirteen male cross bred calves (*Bos taurus* X *B. indicus*) of ten to twelve months old were used in the study. All these cross bred calves were procured at the age of 3-4 months from the Livestock Management Division of the institute and were maintained at tick proof animal rearing facilities of the division. The tick naïve status of the experimental animals was maintained. The experimental animals were maintained as per the approved guidelines laid down by the committee for the purpose of control and supervision of experimentation on animals (CPCSEA), a statutory Indian body.

### 2.3. Laboratory Rearing of *T. annulata* Infection Free *H. a. anatolicum*

The homogenous colony of *H. a. anatolicum* Izatnagar isolate is maintained in the Entomology laboratory of the Division of Parasitology for the last fifteen years [12]. Briefly, healthy New Zealand white rabbits were used for feeding of ticks. To avoid stress on animals, 6–8 rabbits were maintained simultaneously and two rabbits were utilized for each feeding cycle. 

For feeding on large animals, cross-bred male calves were maintained from the initial stage of birth in the tick and fly proof shed of the division of Parasitology. The *T. annulata* free status of the calves were ascertained by periodical examination of Giemsa stained blood smears. 

The engorged *H. a. anatolicum* ticks were collected, identified and were kept in the tick rearing glass tubes covered with mouslin cloth with the help of rubber band. The glass tubes were kept at 28°C temperature and 85% relative humidity (RH) for oviposition. After completion of oviposition, dead females *H. a. anatolicum *were removed from the glass tubes. Larvae were fed on New Zealand white rabbits in which larvae molted to nymph (in rabbit model *H. a. anatolicum* behaved as 2-host tick [12]. The engorged nymphs were collected and maintained. The freshly hatched adults were kept unfed for 7 days and were released on *T. annulata* free male cross-bred calves. The ear bags were checked daily, collected fed adults, cleaned, weighed, labeled and kept singly in the glass tube and were kept in 28°C temperatures and 85% RH for oviposition.

### 2.4. RT-PCR Amplification of Haa86 Gene

Total RNA from the eggs of *H. a. anatolicum* was isolated using the RNeasy total RNA isolation kit (Qiagen). The primers were self designed based on the published sequence information (AF347079). The forward primer (HF2) was designed with BamHI restriction site (HF2-5′CGGC GGATCC TTG TTC GTT GGC GCT ATT TTG CTC AT 3′) and the reverse primer (HR2) was designed with 5′KpnI and XbaI (HR2- 5′CCC GGTACC TCTAGA TGC AAC GGA GGC GGC CAG TAA CAG GA 3′) for subsequent cloning of the PCR product. A 50 *μ*L reverse transcription reaction was set up. Initially, 25.0 *μ*L of total RNA (20 *μ*g) in RNA storage buffer and 0.5 *μ*L of HR2 (100 pm) were mixed in a DEPC treated 0.2 mL PCR tube and kept at 65°C for 10 minutes. To the above mixture 10 *μ*L of Mu-MLV reverse transcriptase buffer (5X)(Invitrogen), 2 *μ*L of RNase inhibitor (2 units)(Invitrogen) and 10 *μ*L of dNTP mix (10 mM each) were added and kept at 37°C for 5 minutes. cDNA synthesis was carried out in the presence of 2 *μ*L of Mu-MLV reverse transcriptase (400 Units)(Invitrogen) at 42°C for 1 hour. The reverse transcriptase enzyme was inactivated by keeping the reaction mixture at 70°C for 10 minutes. A 25 *μ*L PCR reaction was set up using 10X PCR buffer (MBI-Fermentas) containing 2.5 mM of Tris HCl pH 8.3, 2 mM MgCl_2_, 10 mM of each of dNTP, 20 pm of each of the primers HF2 and HR2, 4 *μ*L of the first strand cDNA solution and 2 units of Hot start Taq DNA polymerase (MBI-Fermentas). This mixture was incubated in a thermocycler (PTC-200, MJ Research) with the following cycling conditions: Initial denaturation at 95°C for 5 minutes and further 30 cycles at 94°C for 1 minute, 57°C for 1 minute and 72°C for 2 minutes and a final extension at 72°C for 10 minutes.

### 2.5. Cloning of the Haa86 Gene and Sequencing

Both the PCR product (2 *μ*g) and expression vector pPROEXHTb (Life technologies) were subjected to double restriction enzyme digestion with BamHI and XbaI at 37°C for 6 hours. Both the digested PCR product and plasmid vector were resolved in the 1% agarose and eluted from the gel using Wizard SV Gel and PCR clean up system (Promega) in 50 *μ*L nuclease free water. A 5 *μ*L of the eluted digested PCR product and plasmid vector were resolved in 1% agarose along with DNA ladder 100 bp plus (MBI-fermentas) and concentration of the DNA was calculated by densitometry in the SynGene Gel documentation system. The ligation reaction was set up with 10X ligation buffer (10 mM Tris-HCl, pH 7.5, 10 mM MgCl_2_, 0.1 mg/mL BSA, 0.5 mM ATP), 200 ng of digested vector (pPROEXHTb), 70 ng of digested PCR product and 1 unit of T4 DNA ligase and incubated at 16°C over night. The ligated DNA was transferred into the competent *E. coli* DH5*α* cells treated with 0.1 M CaCl_2_ by heat shock method and the positive clones were screened out based on the blue/white colonies selection and on the ampicillin resistance [13]. Recombinant colonies were confirmed by colony lysis, colony PCR and by plasmid extraction and restriction enzyme digestion. The plasmid with 1965 bp Haa86 gene was designated as pPROHA86F. Both the strands of the insert were sequenced by the dideoxi chain termination method. The nucleotide sequence (ORF) information of Bm86 ortholog of *H. a. anatolicum* Izatnagar isolate (accession no. EU665682) and its deduced amino acid sequence were aligned with the existing sequence information viz., Bm86 ortholog of *H. a. anatolicum* Ludhiana isolate (originated from Punjab state) (accession no. AF347079) and Bm86 of *B. microplus* (Australia) (accession no. M29321) using Gene Tool version 1.0. 

### 2.6. Removal of Signal Sequence and C-Terminal Anchoring Sequence by PCR

The putative signal sequence and C-terminal anchoring sequence were deleted from the ORF of Haa86 by PCR. The forward primer (HF3) was self designed with 5′EcoRI restriction site (GAATTC) (HF3-5′CGGC GAA TTC GGT AGA GAG GAT GAT TTC GTG TG 3′) and the reverse primer (HR4) was designed with 5′XhoI (CTCGAG) (HR4-5′CCC CTC GAG TGT TGC TTC TGT AGT TGT TGC TTC T 3′) for subsequent cloning of the PCR product. A 25 *μ*L PCR reaction was set up using 10X PCR buffer (Ambion) containing 2.5 mM of Tris HCl pH 8.3, 2 mM MgCl_2_, 10 mM of each of dNTP, 20 pm of each of the primers HF5 and HR3, 1.5 *μ*L of 1  : 50 dilution of pPROHA86F DNA (template), 2 units of SuperTaq DNA polymerase (Ambion). This mixture was incubated in a thermocycler with the following cycling conditions: Initial denaturation at 95°C for 5 minutes and further 30 cycles at 94°C for 1 minute, 47°C for 1 minute and 68°C for 2 minutes and a final extension at 68°C for 10 minutes.

### 2.7. Cloning the PCR Product in the Prokaryotic Expression Vector pET32a (Novagen)

The PCR product and the vector pET32a were digested with EcoRI and XhoI. The digested DNA was ligated, transformed and cloned in *E. coli* BL21pLys cells (Novagen). The recombinant or genetically modified *E. coli* BL21pLys was selected based on chloramphenicol and ampicillin resistance. The resulted plasmid construct was designated as pETHA86 and the clones were designated as *E. coli*- pETHA86.PNovagen. The presence of Haa86 gene in the recombinant plasmid extracted from the recombinant *E. coli* clones was confirmed by digesting with restriction enzymes EcoRI and XhoI to release the insert. Subsequently, the presence of insert was also confirmed by colony PCR.

### 2.8. Expression Study

The selected clones were grown for 3-4 hours in the presence of chloramphenicol (34 *μ*g/mL) and ampicillin (100 *μ*g/mL) until the culture attain the absorbance value above 0.6 at 600 nm wavelength. The cultures were induced with 1 mM IPTG and the cultures were grown for 6-7 hours after induction with IPTG. The samples were spun at 10000 X g/10 minutes and the supernatants were resolved in 8% SDS-PAGE along with protein MW marker (Bangalore Genei). The best expressed clone (s) was processed for purification of protein by Ni-NTA affinity chromatography. The induced cultures were pelleted at 10000 X g/10 minutes. The pellets were mixed with lysis buffer (8 M urea, 100 mM NaH_2_PO_4_, 10 mM Tris pH 8.0) and incubated for 1 hour under continuous shaking. The lysates were spun at 10000 X g/10 minutes. The supernatants were added with Ni-NTA resin (Qiagen) 100 *μ*L/1 mL lysate and 10–20 mM imidazole and allowed to bind. The mixture was loaded into small plastic column (Amersham) and was washed with 10 mL washing buffer (8 M urea,100 mM NaH_2_PO_4_, 10 mM Tris pH 8.0). The rHaa86 was eluted with elution buffer (100 mM NaH_2_PO_4_, 10 mM Tris, 8 M urea, pH 4.5).

### 2.9. Determination of the Protein Concentration by Densitometry

The stained band intensity of rHaa86 [14] was compared with the band intensity of different concentrations of BSA resolved in SDS-PAGE. The resolved gels were stored in Syngene gel documentation system and the Syngene software was used for the comparative determination of the protein concentration.

### 2.10. Immunoblotting with Anti-histidine and Anti-Bm86 Antibodies

The appropriate concentration of rHaa86 was resolved in 8% SDS-PAGE under reducing condition and transferred to the PVDF membrane. The membrane strips were separately incubated with primary antibodies (Mouse anti-penta histidine IgG (Qiagen) 1 : 1000 dilution in 1% BSA and rabbit anti-Bm86 antibody (hyperimmune sera) 1 : 150 dilution in 1% skimmed milk in PBST for 2 hours at room temperature. After washing five times in PBST the strips were incubated in secondary antibodies (Anti-mouse IgG- HRPO (Santa Cruz) 1 : 2000 dilution in 1% BSA in PBST and goat anti-rabbit IgG-ALP (Sigma) 1 : 1500 dilution in 1% skimmed milk in PBST, respectively, for 2 hours at room temperature. The membrane strips were washed again with PBST and subsequently incubated in the appropriate substrate solution (10 mL Tris saline (pH 7.6) + 6 mg DAB + 10 *μ*L 30% H_2_O_2_ and 10 mL alkaline phosphatase buffer (pH 9.5) + 100 *μ*L NBT stock solution + 100 *μ*L BCIP stock solution, resp.). The reaction was stopped by placing the membrane strips in distilled water.

### 2.11. Immunization and Challenge Experiment

Cross-bred calves (*n* = 10), aged approximately 7 months, were divided randomly into two groups comprising of five animals in each group. The frozen rHaa86 was emulsified thoroughly with equal volume of adjuvant (10% Montanide 888 in mineral oil). All the animals of group 1 were inoculated with 400 *μ*g of rHaa86 intramuscularly on day 0, 400 *μ*g on day 30 and 100 *μ*g on day 60. The corresponding control animals were inoculated with equal volume of adjuvant on the same day. Each calf (groups 1, 2) was challenged on day 97 postimmunization with fifty uninfected adults of both sexes (male and females in 1 : 1 ratio) by ear bag method. Following post challenge entomological parameters were recorded [15].

- The number of engorged adult female ticks dropped from each animal was recorded.
- Dropped engorged ticks were weighed individually.
- The engorged female ticks were incubated for laying eggs and the egg masses were weighed.
- DT% = 100(1−NTV/NTC), Where DT% is the percentage reduction of females, NTV, the number of females dropped from the animals of group 1 and NTC, the number of females dropped from the animals of group 2.
- DO% = 100(1−PATV/PATC), where DO% is the percentage reduction of mean weight of eggs, PATV the mean weight of eggs of females fed on animals of group 1 and PATC the mean weight of eggs of females fed on animals of group 2.
- DR% = 100(1−PMTV/PMTC), where DR% is the percentage reduction of mean weight of adult females, PMTV the mean weight of adult females dropped from the animals of group 1, and PMTC the mean weight of adult females dropped from the animals of group 2.
- E% =  100[1−(CRT × CRO)], Where E% is the efficacy of antigen, CRT is the reduction in the number of adult females NTV/NTC, CRO is the reduction in egg laying capacity, PATV/PATC (PATV, the mean weight of eggs of ticks fed on the animals of group 1)/PATC, the mean weight of eggs of females fed on animals of group 2.

### 2.12. Enzyme Linked Immunosorbent Assay

Blood samples were collected aseptically from all the calves during pre and post tick challenge periods in a regular interval. Sera were separated, aliquoted and stored at −20°C. Initially checkerboard titration was used to optimize the reagents. After optimization, the eluted antigen was applied to the microtire plate (Nunc) in a concentration of 4 *μ*g/mL and kept at 40°C for overnight. After washing thrice with PBST, the wells were blocked with 5% nonfat milk in PBST for 2 hours at RT. After three washes, primary antibodies were diluted 1 : 50 in 1% PBST and were used in quadriplicate wells and the plates were kept at RT for 2 hours. After washing, the secondary antibody (anti-bovine peroxidase conjugate, Sigma Chemical Company, USA) was used at a dilution of 1 : 10000 in 1% PBST for 2 hours. The reaction was stopped with 50 *μ*L 3N HCl per well, and absorbance was recorded by microplate ELISA reader (Tecan-Sunrise, Austria), as the mean OD_492_ of triplicate samples.

### 2.13. Statistical Analysis

Significant differences in mean values from immunized and control animals were determined using student's *t*-test [16].

## 3. Results

### 3.1. Construction of Bacterial Expression Vector with Haa86 Gene Fragment

**Figure 1:**
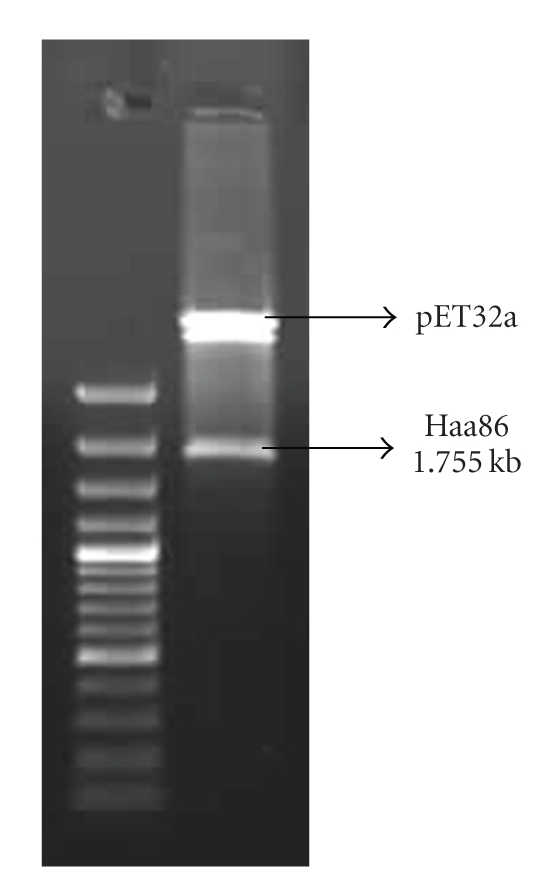
Release of insert after restriction digestion with EcoRI and XhoI. The 1.755 bp Haa86 gene was released by the restriction enzyme digestion reaction.

**Figure 2:**
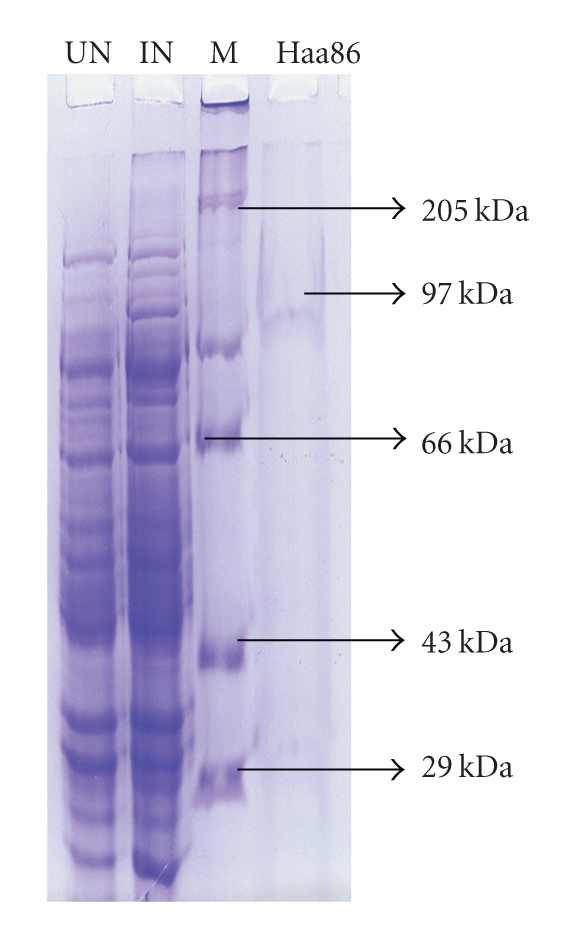
The SDS-PAGE profile of rHaa86. Legends: UN-uninduced, IN induced, M-molecular weight marker, Haa86-97 kDa expressed protein.

The size of the Haa86 gene fragment amplified by RT-PCR with primers HF2 and HR2 was 1965 bp. After confirmation of gene, the fragment was cloned into pPROEXHTb vector to obtain the construct pPROHA86F. Sequence lengths of 144 bp from 5′ end and 96 bp from 3′ end were deleted from the ORF of the Bm86 ortholog of *H. a. anatolicum* by performing PCR with primers HF3 and HR4. The shortened Haa86 ORF with the size of 1755 bp was cloned in expression vector and the resultant plasmid construct was designated as pETHA86 and the clone was designated as *E. coli*-pETHA86. The presence of Haa86 gene in the recombinant plasmid extracted from the recombinant *E. coli* clone was confirmed by digesting with restriction enzymes EcoRI and XhoI to release the insert. The 1.755 bp gene was released by the restriction enzyme digestion reaction (Figure 1). Recombinant Haa86 was expressed in vitro using pET-32a expression vector amd the *E. coli* strain Bl21(DE3)pLysS expression system. Affinity purified rHaa86 migrated as 97 kDa protein on 8% SDS-PAGE, consistent with the expected molecular mass considering that the expression vector produced a recombinant protein fused with a 19 kDa thioredoxin protein (Figure 2). Upon purification by Ni-NTA resin, rHaa86 seemed to be more than 98% pure as observed in SDS-PAGE.

**Figure 3:**
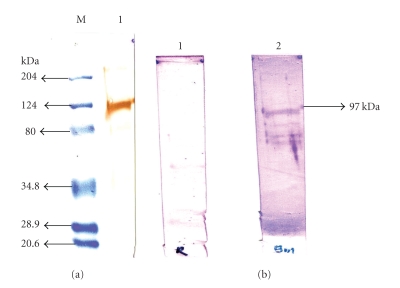
Immunoblot showing reaction of rHaa86 with (a) mouse antipenta histidine antibody; lane 1, strong reaction with rHaa86 and lane M, molecular weight marker and (b) with anti-Bm86 antibody; lane 1, no reaction with normal sera and lane 2, reaction with anti-*H. a. anatolicum* larval antibody.

**Figure 4:**
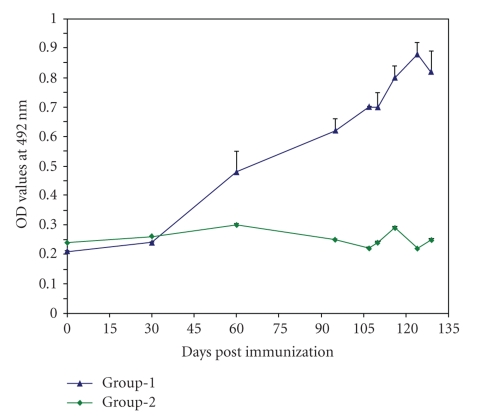
Antibody response in calves immunized with rHaa86 (Gr. 1) and control animals (Gr. 2).

When the transferred protein was probed with mouse antipenta histidine antibodies, the anit-histidine antibodies reacted strongly with rHaa86 around 97 kDa (Figure 3). Similarly, anti-Bm86 antibodies reacted strongly with rHaa86 protein. No reaction was noted when control rabbit sera was used to probe the PVDF containing rHaa86 (Figure 4). Positive signals were also obtained at lower molecular proteins, a possible degraded product, than that of putative rHaa86. This result demonstrates the cross reactive nature of the Bm86 of *B. microplus* and its ortholog in *H. a. anatolicum*.

### 3.2. Analysis of the Sequence Information

Identity of this Bm86 ortholog of *H. a. anatolicum* Izatnagar isolate (EU665682) with the *H. a. anatolicum* Ludhiana isolate (AF347079) was 99.3% and 98.7% at nucleotide sequence and deduced amino acid sequence level, respectively. The identity with Bm86 of *B. microplus* (Australia) (M29321) was 76.1% and 60.9% at nucleotide sequence and deduced amino acid sequence level, respectively. Eight amino acid substitutions were observed in the deduced amino acid sequence of Haa86 when compared to Ha98 sequence. Amongst the eight, five amino acid substitutions were nonconservative in nature. The 20th, 47th, 290th, 421st and 438th amino acids of Haa86 were substituted with glutamic acid (E), glycine (G), lysine (K), alanine (A) and aspartic acid (A), instead of lysine (K), arginine (R), asparagine (N), glutamic acid (E) and glycine (G), respectively.

### 3.3. Feeding and Reproductive Performances of *H. a. anatolicum*

In all the animals, the 7–10-days-old unfed adults started feeding on all the animals within 48 hours of their release. After 120 hours of challenge, a mean number of 7.4 ± 1.9 female ticks dropped from the animals of group 1 while 28.3 ± 5.0 ticks dropped from the animals of group 2 and the difference in the number of ticks dropped from the immunized and control group of animals was found statistically significant (*P* < .01). A significant (*P* < .01) mean reduction of 49.6 mg in the weight of ticks fed on group 1 animals in comparison to the ticks fed on group 2 animals was noted. The dropped ticks were kept for oviposition and a mean reduction of 68.1 mg (*P* < .05) in the egg masses laid by the ticks fed on immunized animals in comparison to the ticks fed on group 2 animals was noted (Table 1). The direct effect of immunization (DT%) on the number of female was 73.8%. The other entomological parameters DO%, DR% and E% were calculated as 31.3, 15.8 and 82.3%, respectively.

### 3.4. Antibody Response in Calves

In group 1 animals, a significantly high anti-Haa86 antibody responses in comparison to group 2 animals were detected after first boosting and antibody responses reached at peak on 124 days of first immunization (dfi) (*P* < .001). At the time of challenge (97 dfi), the antibody responses in calves of group 1 increased significantly (*P* < .01) and the anti-Haa86 antibodies interfered with the feeding and reproductive efficiency of ticks fed on immunized animals (Figure 4). Till 129 dfi the antibody response was maintained at a significantly high level.

## 4. Discussion

In our continuous effort to develop vaccine against *H. a. anatolicum*, progress has been made to identify native proteins of vaccine potential [9]. Recently, the Bm86 ortholog gene of *H. a. anatolicum* (Haa86) has been cloned in pBluescript II KS for subsequent expression in *P. pastoris* expression vector GS115 (*his4*) pPICZ*α*A [11]. The structure of reading frame in the expression cassette consisted of N terminal *α*-factor followed by 1799 bp Haa86 gene with 26 bp from pBluescript II KS (+) and 73 bp sequence from pPICZ*α*A that includes c*myc* epitope and 6 X histidine tag. The total size of the coding sequence was 1872 bp. The linearized pPICZHA86 had free ends homologous to 5′*AOX*1 promoter region and 3′*AOX*1 transcription termination sequence of *P. pastoris*, to help in directing the integration of the expression cassette into the *AOX*1 locus of the *P. pastoris* genome by site specific homologous recombination resulting in the substitution of the endogenous AOX1 structural gene. The recombinant Haa86 was successfully expressed in the methyl tropic yeast but was unsuccessful in the purification of the *P. pastoris* expressed rHaa86 by Ni-affinity chromatography. The purification of the rHaa86 was done by exploiting the particulate nature of the protein as like that of the purification of *P. pastoris* expressed Bm86 with some modifications [17, 18].

Although the protein has expressed successfully in GS115 (*his4*) pPICZ*α*A, the level of expression was not satisfactory. Probably the GS115 strain is not suitable for expression of the Haa86 gene. Similar type of problems was noted with other parasitic genes viz., VSG of *Trypanosoma evansi*. Besides, a number of steps viz., disruption, centrifugation, washing, extraction, refolding, precipitation and ultra filtration were involved in the purification process. During the long purification process a significant amount of protein has been lost and ultimately the recovery was considerably low. In the present experiment, the rHaa86 protein was expressed as fusion protein and was purified by affinity chromatography involving minimum steps and thus minimized the loss during processing. The level of expression was 40% more than the *P. pastoris* system. Besides the minimum steps involved in the purification process, the purification protocol gave comparatively higher level of purification (more than 98%) than has been done previously [11]. The recovery percentage of rHaa86 expressed in *E. coli* was comparatively higher (3.0 mg/Litre) than the *P. pastoris* expressed protein (0.8–1.0 mg/Litre) [11].

Recognition of rHaa86 by the anti-histidine antibodies in immunoblotting indicates the expression of rHaa86 to its full length. The reaction of anti-Bm86 polyclonal antibody with the rHaa86 indicates the cross reactive nature of the Bm86 with Haa86. Saimo et al. [19] reported the cross-reaction of the anti-Bm86 antibodies with Ra86 expressed in insect cell system (Bm86 homologue of *Rhipicephalus appendiculatus*). These studies suggest the conservation of sequential epitopes between the Haa86, Bm86 and Ra86. 

The present experiment is the second immunization and challenge trial using the recombinant antigen of *H. a. anatolicum*, rHaa86. Therefore, the entomological parameters were compared with immunization trial of Bm86 vaccine (Gavac) against *B. microplus* and *H. a. anatolicum *and the results obtained during first immunization trial. The rejection percentage, reproductive index, DT% and E% of the rHaa86 immunized animals against adult *H. a. anatolicum* are highly encouraging in nature. The E% of the Gavac vaccine against different strains of *B. microplus* showed 51% to 91%. Amongst the tested *B. microplus* strain, half of the strains were showing E% of 51 to 60 and half of the strains were showing 72 to 91. These results were obtained by challenging the immunized calves with *B. microplus* larvae [18]. In the present experiment, 72.0% efficacy was obtained by challenging the immunized calves with adults of *H. a. anatolicum*. The E% value of the present study is falling within the range of different experiments conducted using Bm86 based vaccine. The DT% of the Gavac vaccine against different strains of *B. microplus* was 9% to 74%. Amongst the ten strains, only two of the strains were showing DT% above 50%. The comparatively high DT% of 73.8% obtained in the present experiment is highly encouraging and was falling within the range of commercialized vaccine Gavac [18].

When the immunoprotective properties of the glycosylated and nonglycosylated rHaa86 were compared, it was observed that DT%, DR%, DO% and E% of ticks fed on animals immunized with glycosylated Haa86 were 58, 9, 5 and 61.6%, respectively, while the corresponding reduction percentages of biological parameters of ticks fed on animals immunized with nonglycosylated rHaa86 were 73.8, 15.8, 31.3 and 72.0, respectively. The results indicated that glycosylation is not important to provide significant protection against *H. a. anatolicum*. For Bm86 antigen, the glycosylation, which may represent about a third of the native protein, is highly immunogenic but appears not to be important for protective immunity [20]. Recombinant Bm86, whether expressed in *E. coli*, insect cells or *P. pastoris* appears to be about as efficacious as native antigen [21]. In contrast, Lee et al. [22] reported that the destruction of protective activity by periodate treatment strongly suggests that all protection was due to carbohydrate epitopes. It may be concluded that the importance of glycosylation to protective antigenicity needs to be assessed on a case to case basis.

The significantly high level of reduction of entomological parameters of ticks fed on group 1 animals in comparison to the ticks fed on control animals has a direct effect on the reduction of population of the tick species in the environment, which in turn will definitely minimize the tick load on animals.

## Figures and Tables

**Table 1:** Feeding and reproductive performances of ticks fed on immunized and control calves.

Experimental calves	Mean values ± SE		
	Mean no. of ticks dropped	Mean wt. of engorged adults (mg)	Mean egg masses (mg)
Group 1 (Immunized)	7.4 ± 1.9^a^	263.2 ± 10.9^a^	149.3 ± 13.0^b^
Group 2 (control)	28.3 ± 5.0	312.8 ± 9.3	217.4 ± 19.5

^a^ *P* < .01; ^b^ *P* < .05.
